# Big data in global health: improving health in low- and middle-income countries

**DOI:** 10.2471/BLT.14.139022

**Published:** 2015-01-30

**Authors:** Rosemary Wyber, Samuel Vaillancourt, William Perry, Priya Mannava, Temitope Folaranmi, Leo Anthony Celi

**Affiliations:** a Telethon Kids Institute, University of Western Australia, 100 Roberts Road, Subiaco, Western Australia, Australia.; b Li Ka Shing Knowledge Institute of St Michael's Hospital, Toronto, Canada.; c Nyes Institute, Christchurch, New Zealand.; d Division of Health Science and Technology, Harvard-MIT, Cambridge, United States of America.

## Abstract

Over the last decade, a massive increase in data collection and analysis has occurred in many fields. In the health sector, however, there has been relatively little progress in data analysis and application despite a rapid rise in data production. Given adequate governance, improvements in the quality, quantity, storage and analysis of health data could lead to substantial improvements in many health outcomes. In low- and middle-income countries in particular, the creation of an information feedback mechanism can move health-care delivery towards results-based practice and improve the effective use of scarce resources. We review the evolving definition of big data and the possible advantages of – and problems in – using such data to improve health-care delivery in low- and middle-income countries. The collection of big data as mobile-phone based services improve may mean that development phases required elsewhere can be skipped. However, poor infrastructure may prevent interoperability and the safe use of patient data. An appropriate governance framework must be developed and enforced to protect individuals and ensure that health-care delivery is tailored to the characteristics and values of the target communities.

## Résumé

Au cours de la dernière décennie, une augmentation massive de la collecte et de l'analyse des données s'est produite dans de nombreux domaines. Dans le secteur de la santé, cependant, il y a eu relativement peu de progrès en matière d'analyse et d’application des données malgré une hausse rapide de la production des données. Grâce à une gouvernance adéquate, les améliorations en matière de qualité, de quantité, de stockage et d'analyse des données de santé pourraient se traduire en des améliorations substantielles pour de nombreux résultats cliniques. Dans les pays à revenu faible et intermédiaire en particulier, la création d'un mécanisme de retour d'information peut faire évoluer la prestation des soins de santé vers une pratique axée sur les résultats et améliorer l'utilisation efficace des ressources limitées. Nous examinons l'évolution de la définition des mégadonnées («big data») et les avantages – et les problèmes – possibles de l'utilisation de ces données pour améliorer la prestation des soins de santé dans les pays à revenu faible et intermédiaire. Alors que les services basés sur la téléphonie mobile s'améliorent, la collection des mégadonnées peut signifier que les phases de développement requises ailleurs peuvent être omises. Cependant, une infrastructure faible peut empêcher l'interopérabilité et l'utilisation en toute sécurité des données du patient. Un cadre de gouvernance approprié doit être développé et appliqué pour protéger les individus et assurer que la prestation des soins de santé est adaptée aux caractéristiques et aux valeurs des communautés cibles.

## Resumen

En la última década, la recolección y análisis de datos ha aumentado enormemente en muchos campos. Sin embargo, en el sector de la sanidad ha habido relativamente pocos avances en el análisis y la aplicación de datos, a pesar de un aumento rápido en la generación de datos. Con una gestión adecuada, las mejoras en la calidad, cantidad, almacenamiento y análisis de datos de salud podrían conducir a mejoras considerables en muchos de los resultados sanitarios. En los países de ingresos bajos y medios en particular, la creación de un mecanismo de retroalimentación de información puede impulsar la prestación de servicios sanitarios hacia la práctica basada en resultados y mejorar el uso eficaz de los recursos escasos. Se revisó la definición cambiante de los grandes volúmenes de datos y las posibles ventajas – y problemas – de utilizar dichos datos para mejorar la prestación de servicios sanitarios en países de ingresos bajos y medios. La recogida de grandes volúmenes de datos a medida que mejoran los servicios basados en la telefonía móvil podría permitir la omisión de las fases de desarrollo requeridas en otros lugares. Sin embargo, una infraestructura deficiente puede impedir la interoperabilidad y el uso seguro de los datos del paciente. Es necesario desarrollar y aplicar un marco de gestión adecuado para proteger a las personas y garantizar que la prestación de servicios sanitarios se adapta a las características y los valores de las comunidades destinatarias.

## ملخص

شهد العقد المنصرم زيادة هائلة في جمع البيانات وتحليلها في العديد من المجالات. لكن قطاع الصحة، لم يشهد سوى تقدماً ضعيفاً نسبياً في تحليل البيانات وتطبيقها على الرغم من الارتفاع السريع في إنتاج البيانات. وبالنظر إلى تصريف الشؤون الملائم، من الممكن أن تؤدي التحسينات في جودة البيانات الصحية ومقدارها وتخزينها وتحليلها إلى تحسينات جوهرية في العديد من الحصائل الصحية. وفي البلدان المنخفضة الدخل والبلدان المتوسطة الدخل، من الممكن أن يؤدي إنشاء آلية للتغذية المرتدة للمعلومات إلى نقل إيتاء الرعاية الصحية نحو الممارسة المستندة إلى النتائج وتحسين الاستخدام الفعال للموارد الشحيحة. ونستعرض التعريف المتطور للبيانات الضخمة والمزايا المحتملة لاستخدام هذه البيانات ومشكلاتها بغية تحسين إيتاء الرعاية الصحية في البلدان المنخفضة الدخل والبلدان المتوسطة الدخل. وقد يعني جمع البيانات الضخمة في الوقت الذي تتحسن فيه الخدمات المستندة إلى الهواتف المحمولة إمكانية تخطي مراحل التطوير المطلوبة في أماكن أخرى. ومع ذلك، قد يمنع ضعف البنية الأساسية التشغيل البيني والاستخدام الآمن لبيانات المرضى. ويجب تطوير إطار ملائم لتصريف الشؤون وإنفاذه لحماية الأفراد وضمان إيتاء الرعاية الصحية المخصصة وفق خصائص المجتمعات المستهدفة وقيمها.

## 摘要

过去十年，许多领域的数据收集和分析已大量增加。然而，在卫生部门，尽管数据生产快速增长，数据分析和应用的发展却相对较缓。适当管理健康数据、改善其质量、数量、存储和分析可能有助于明显改善许多健康状况。特别在中低收入国家，建立信息反馈机制可以将医疗交付转变为基于成果的实践，提高稀缺资源的有效利用。我们回顾了不断演进的大数据定义，以及使用这些数据来改进中低收入国家的医疗保健可能的优点和问题。随着基于手机服务的改善，大数据收集可能意味着可以跳过其他地方所需的发展阶段。然而，落后的基础设施可能妨碍病人数据的互操作性和安全使用。应发展并实施适当的治理框架，以便为保护个人提供保护，并确保提供完全符合目标社区特征和价值观的医疗保健。

## Резюме

В последнее десятилетие во многих областях отмечался резкий рост сбора данных и их анализа. Однако в секторе здравоохранения наблюдался относительно небольшой прогресс в анализе и применении данных, несмотря на быстрое увеличение объема генерируемых данных. При наличии соответствующего управления, улучшение качества, количества, хранения и анализа данных о здоровье населения могло бы привести к существенному улучшению многих результатов медицинских мероприятий. В частности, в странах с низким и средним уровнями доходов создание механизма обратной связи может сделать предоставление медицинских услуг процессом, в большей степени ориентированным на конкретные результаты, и повысить эффективность использования ограниченных ресурсов. Был проведен обзор эволюции определения больших данных, а также возможные преимущества и проблемы использования подобных данных для улучшения предоставления медицинских услуг в странах с низким и средним уровнями доходов. Сбор больших данных по мере совершенствования услуг на базе для мобильных телефонов может означать возможность пропуска этапов развития, обязательных в других местах. Однако слаборазвитая инфраструктура может препятствовать совместимости и безопасному использованию данных о пациентах. Должна быть разработана и внедрена соответствующая система управления, чтобы защитить физических лиц и обеспечить адаптацию медицинских услуг к характеристикам и ценностям целевых сообществ.

## Introduction

The delivery of health care is a complex endeavour at both individual and population levels. At the clinical level, the tailored provision of care to individuals is guided, in part, by medical history, examination, vital signs and evidence. In the 21st century these traditional tenets have been supplemented by a focus on learning, metrics and quality improvement. The collection and analysis of data of good quality are critical to improvements in the effectiveness and efficiency of health-care delivery. A substantial fraction of the waste in health-care expenditure results from not knowing what works for particular patients in particular clinical contexts. Interventions that appear effective in population-based studies are often widely implemented without any monitoring or any attempt to identify the patients more likely to benefit from – or to be harmed by – the interventions. The challenges of generating, analysing and applying clinical data are particularly acute in low- and middle-income countries. Given the sheer size of the human population and the incredible complexity of health-care delivery – with thousands of diseases and thousands of medications and interventions – the reconciliation of data-driven improvements in clinical medicine with good population health is complex. However, the recent development of new methods to collect, analyse and apply data on an unprecedented scale – the so-called big data approach – may allow the gap between health-care delivery and population health to be bridged and many health outcomes to be improved. These new methods of collecting, curating and conceptualizing numbers offer similar advantages to populations as those offered to patients by medical imaging – i.e. they offer the opportunity to see with greater precision.

In many fields, over recent decades, the generation and use of data have rapidly expanded and new data applications have been developed. However, although there has been a concurrent increase in the production of data in the health sector, there has been little corresponding change in the use of such data to improve health care.^1^ Some proof-of-concept applications have been created for – and enthusiastically received by – health professionals but, so far, they have resulted in very few tangible, system-wide data initiatives. In this article we explore some current and potential applications of big data to public health and health-care delivery in low- and middle-income countries. We searched the PubMed and Embase databases and the grey literature for relevant articles and checked the references of selected articles for other sources of relevant information. We explored the benefits, risks and opportunities for big data in health and made recommendations for the use of big data in the delivery of health-care services in low- and middle-income countries. The multiple applications of big data to genomics and life sciences have been widely reviewed elsewhere and are not addressed in this article.

## Definition

The term big data refers to the emerging use of rapidly collected, complex data in such unprecedented quantities that terabytes (10^12^ bytes), petabytes (10^15^ bytes) or even zettabytes (10^21^ bytes) of storage may be required.^2^ The unique properties of big data are defined by four dimensions: volume, velocity, variety and veracity.^3^ As more information is accruing at an accelerating pace, both volume and velocity are increasing. Use of a variety dimension marks a shift from data as information that is collected directly to information that is assimilated from multiple sources. Big data outputs tend to increase in value as sources become more diverse. At a population level, traditional health data included information from vital statistics registries and hospital admission statistics. In the last few decades, however, more health data have been assimilated from electronic medical records, mobile phone and purchase records, geographical positioning systems, social media and beyond. The veracity dimension refers to the uncertainty around data and their collection, standardization and validation. As the quantification and articulation of the uncertainty in reported data have been a part of health-care research and practice for many years, health practitioners may be more familiar with data veracity than many other users of big data. Uncertainty and confidence intervals are now commonly reported in projects that use these data. Together, these dimensions enable a big data approach to health, in which health priorities and policies are driven by analytics of large data sets. 

## Applications

The analysis of linked data sets from different sectors can provide new opportunities to improve health outcomes for populations. In the United States of America, for example, health-care and city authorities in Durham County, North Carolina, decided to pool data to direct social and health care. They created an integrated data system that allowed for the coordination of efforts to improve emergency department services and the care of patients with diabetes.^4^ By geographically pooling census data, tax payments and lead concentrations detected in blood tests, it was also possible to use the integrated system to map and stratify risks of lead exposure. Screening for lead exposure could then be focused on the high-risk areas and this led to vast improvements in the detection and management of childhood lead exposure.^4^ Such sharing and analysis of information can bridge the chasm that has traditionally divided population health from clinical medicine at individual level.

The early adoption of the big data approach in well resourced settings highlighted some logistical, technical, ethical and governance challenges. In Iceland in 1998, for example, the health records – including genetic data – were declared to be a national resource by the government and made available to private industry without the consent of individuals.^5^ However, national and international opposition prevented any data transfer and the project had collapsed by 2003.^5^ Best et al. stated that “outside the world of carefully-controlled trials, between 50 and 80 per cent of electronic patient record projects fail”.^6^ Petabytes of health data have been collected, but have not been used.^7^

Although the use of big data in low- and middle-income countries is particularly complex, it also offers the greatest potential rewards. Most such countries have vertical programmes for the control of human immunodeficiency virus, tuberculosis, malaria and other infections. These programmes have detailed information requirements that need to be met by community health workers. There is often a mismatch between the information needs of the programme and the capacity of the associated field personnel to collect data with sufficient quality for reporting, tracking and – more importantly – learning. However, the advent of electronic tools is circumventing some of the logistic and quality issues in data collection.^8^ Community health workers can use mobile phones, tablets and computers for research and patient care.^9^^,^^10^

One of the most promising examples of big data in global health may emerge from India’s ambitious personal identification programme. Since 2010 the government of India has been issuing Aadhaar cards and unique identifying numbers to all 1.2 billion of its citizens.^11^ The cards, numbers and associated biometric identification offer the possibility of generating and monitoring health and social data – including electronic medical records and information on health insurance for low-income families – on a huge scale.^12^^,^^13^ Even if limited in its current reach, the Aadhaar system forms a backbone that could allow the more reliable and extensive collection of health statistics. This, in turn, could lead to dramatic improvements in the planning and delivery of public health interventions.

Recent cross-sectoral data sharing is stimulating rapid innovation and growth. In 2013, for example, the government of Côte d’Ivoire consented to the release of five months’ of anonymized mobile phone data. These data were then used to develop a model for containing the spread of epidemics.^14^ This kind of data philanthropy or liberation – in which corporate entities or governments share anonymized information of potential public health significance – may provide new synergies between the for-profit sector and other stakeholders.^15^

## Use

The diffusion of new technology in low- and middle-income countries is commonly a mix of appropriation, diffusion and, often, the skipping of some of the intermediate development phases observed in high-income countries. One example of leapfrogging is the penetration of mobile phones – before the development of a widespread wired phone system – in low-resource settings over the past 15 years. The opportunities offered by the many mobile phones in such settings in the improvement of health delivery have spurred widespread enthusiasm for so-called mobile health – or m-health – projects.^16^ Several small-scale m-health programmes have demonstrated proof of concept, but few of the tested interventions have been implemented on a large scale.^17^

There are differences between the typical m-health and big data approaches. In general, m-health projects are relatively democratized, have low barriers to entry and capitalize on the mobile phones already owned by individuals. Although the impact of such projects is often rapidly appreciable, the tangible rewards are often very limited.^18^ In contrast, the big data approach inherently demands more technical skills, specialized equipment, interoperability standards, coherent data collection and analysis systems and regulatory oversight. Beyond the technical aspects, an organizational culture of quality is one of the key drivers of an effective health information system. Health-care providers and system administrators in most countries have not been trained in data science. 

To support good data collection, interoperable information systems based on fixed standards also need to be put in place. Global norms should be established before the highly regulated and path-dependent legacy systems found in high-income countries become fragmented in low and middle-income settings. In the smart use of data to support implementation science, initiatives such as the results-focused approach to data analysis and sharing implemented at the high-volume, low-cost Narayana Hrudayalaya Heart Hospital in India have improved health outcomes.^19^ Although the pooling of data across systems presents some major obstacles, there are emerging signs of data ownership in some low- and middle-income countries. One such sign is the launch of a repository to collate health data.^20^

## Challenges

There are many challenges to the full-scale implementation of big data systems in low- and middle-income countries. The collection of information from individuals – a prerequisite for any big data approach – is fraught with ethical, regulatory and technological issues. Given the increasing complexity of the field, the protection of individuals and populations must move from purpose-specific consent to emphasize appropriate use, risk assessment and risk minimization. The anonymization of data must be robust, monitored and enforced. Appropriate use must remain coherent with evolving societal values. Furthermore, the big data approach can amplify the existing difficulties associated with health-care delivery in settings with scarce resources. In such settings, it may be impossible for front-line health workers to extend their remit to the non-essential collection of data. Some policy-makers view the big data approach simply as a distraction for low- and middle-income countries.^15^ Others consider big data to be a critical milestone on the path towards the improvement of such countries (Box 1).

Box 1 **Differing views on the big data approach**

In low- and middle-income countries, the future could go well or badly for the big data approach.

**Dystopian views**

In the worst-case scenario, big data would be an expensive distraction driven by high-income countries, focused on disease-specific outcomes and unintelligible to those who most need data access. The assimilation of fragmented data – which cannot be readily shared or compared – could undermine the relatively fragile global health community. Breaches of data security could threaten personal safety and lead to discrimination and genocide and other violence. The global health community could oversee the spending of huge amounts of money on big data, with potentially little to show for the investment.

In brief, the big data approach could be associated with:

- the diversion of focus and resources away from interventions that are more needed;
- poor data governance – with databases held by private companies, frequent leaks and no recourse for citizens;
- the offloading of consent through poorly designed consent systems, which could threaten the safety of individuals;
- a lack of interoperability, with balkanized information systems that cannot be aggregated; and
- information that is poorly presented and analysed, considered illegible or not credible.

**Utopian views**

Conversely, the big data era could represent a major and beneficial turning point in the improvement of global health. Decision-makers in low- and middle-income countries could develop a “demand-side” platform to identify the information they need most. Partnerships formed with academia, industry, governments, international organizations and the non-profit sector could help develop innovative solutions. Although this idealized approach is optimistic, it is no less ambitious than achieving the Millennium Development Goals, eradicating polio or controlling malaria. The development of a “best-case” model for deploying big data may help us achieve all of these targets.

In brief, the big data approach could be associated with:

- health data that are owned by patients;
- robust governance processes that have been developed to ensure respect of values and principles in the use of data, with an emphasis on risk minimization;
- data that are aggregated automatically, with little effort and decreasing cost;
- interoperability standards that allow data to be seamlessly pooled and connected;
- laws that, while establishing adequate safeguards, allow the sharing and pooling of anonymized data in real time; and
- data that are presented in a usable format to patients, health-care providers, entrepreneurs and policy-makers.

Even in the best of cases, threats to the privacy of personal health information will remain. This concern is amplified when the information relates to individuals in vulnerable populations and communities. Even basic health data – e.g. on ethnicity, reproductive health, sexually transmitted infections, diseases with a genetic basis and risk exposures for disease – can be misused and lead to discrimination and reductions in personal safety.^21^ Any electronic database can be hacked. The risk of accidental or intentional breaches of data security may be particularly high in settings with high levels of illiteracy and corruption that are undergoing rapid technological transition.^22^ In many such settings, legislation supporting the privacy and security of information services is frequently underdeveloped and rarely enforced.

Even in high-income countries, purpose-specific informed consent is increasingly being rendered meaningless by high levels of complexity in the ways that collected data are – or might be – used. Privacy protection is a right and the preservation of public trust is a necessity. However, as the full potential of the big data approach to improve health becomes clearer, there is also a right for populations to reap all of the potential benefits of such an approach.^23^ The use of anonymized data for the greater good of populations needs to be incorporated into the process of risk minimization. There is an increasing need for traditional consent protocols to be replaced by – or supplemented with – transparent and effective processes for data governance. The values and concerns of the target populations need to be translated into best practices that balance the benefits and risks of data use. Concerns persist about data sharing and appropriate use.^24^

The promise of big data is tempered by the weak health systems and limited governance structures to be found in most low-income countries. Many of the countries in greatest need of health metrics struggle to collect statistics on births and deaths. The epidemiological data collected in these countries are of variable reliability, have often only been collected at small sentinel sites and are rarely digitized. Improvements in the provision of food, water and sanitation remain the top priorities for over two billion people. In many low-income countries, data collection may only be possible at the expense of tangible health services. As reported by the United Nations, “it is important to recognize that big data and real-time analytics are no modern panacea for age-old development challenges”.^15^ However, as the cost decreases of aggregating and coordinating resources and services electronically, the big data approach may deliver large benefits to low- and middle-income countries. The more limited the resources for interventions, the more important the targeting and focusing of interventions become.

The persistent tension between vertical or disease-specific programmes and horizontal or health-system-focused approaches remains unresolved. The big data approach fits a horizontal programme better than a vertical programme and could potentially improve the control and treatment of all human disease. At the moment, global health remains driven by disease-specific interests and disease-specific advocacy groups may well head the queue for big data – risking further fragmentation of the health community.

## The next step

The role of big data collection – whether it is perceived as a tool or a threat – remains unclear. For positive outcomes, informed, reflective and resourced stewardship of data is critical. At the moment, the structures for global health governance remain relatively fragile.^25^ In 2009, the United Nations established the Global Pulse initiative. “Its mission is to accelerate discovery, development and scaled adoption of big data innovation for sustainable development and humanitarian action.”^26^ Unfortunately, the current data protection standards for Global Pulse are badly outdated as they are grounded in guidelines – for the regulation of computerized personal data files – that were published in 1990.^27^

Some guidance on the collection and use of health data was provided within the World Economic Forum’s *Global health data charter*, as part of the Forum’s vision of “better data for better health”.^28^ For health data, the charter identified eight key challenges and highlighted several enabling activities. The expansive scope of big data requires the cooperation of multiple stakeholders. Universities, professional societies, government agencies and research-driven companies are examples of organizations that could develop and operate data systems to support health care. A clear governance and decision-making framework is needed to inform each stakeholder of its accountability and responsibility for each process. There needs to be transparency in addressing and troubleshooting any issues until major decisions are made. Issues often persist for lack of ‎clear agreements on who should resolve them and how they should be resolved. In an emerging field such as big data, where protocols are still being developed, governance plays a major role in assuring stakeholders that there is a system for resolving issues.

However, the global health community has a patchy record on governance of technological developments.^29^ Optimizing the application of big data will involve much more than confidentiality safeguards and minimum standards. A broad effort to establish interoperability standards is imperative to maximize the benefits of big data. Global health governance needs to move from a reactive model to a proactive, norm-forming approach.

## Conclusion

In the field of health-care delivery, the big data approach may represent a major milestone – facilitating the development of learning systems of care and enabling more precise management of individuals to improve the health of entire populations. Sheer size increases both the potential risks and potential benefits of the approach. Although the approach may have most value in low-resource settings, it is also most vulnerable to fragmentation and misuse in such settings. Collaborative governance, careful analysis and technical partnerships are needed to minimize the risks. The complexities should not be underestimated. In low- and middle-income countries, the shepherding of the transition from paper records to petabytes of digital storage provides another opportunity for global health institutions to offer useful governance.
